# Stauffer’s Syndrome in Pancreatic Cancer: First Case Report

**DOI:** 10.7759/cureus.1230

**Published:** 2017-05-08

**Authors:** David Harris, Muhammad W Saif

**Affiliations:** 1 Internal Medicine, Tufts Medical Center; 2 Hematology/Oncology, Tufts Medical Center

**Keywords:** pancreatic cancer, stauffer's syndrome, abnormal liver functions, metastases, paraneoplastic syndrome, hypercoagulability

## Abstract

Stauffer’s syndrome is a rare paraneoplastic syndrome classically associated with renal cell carcinoma. It presents as abnormal hepatic panel in the absence of hepatic disease, which improves with treatment of the cancer and worsens with recurrence. Here, we describe a case of hepatic panel abnormalities in a patient with pancreatic cancer with no evidence of metastatic disease to the liver, primary hepatobiliary etiology, or clear offending medications. We believe this to be the first reported case of Stauffer’s syndrome in patients with pancreatic cancer.

## Introduction

Pancreatic cancer is the fourth leading cause of cancer-related deaths in North America, with approximately 53,000 patients diagnosed annually. The majority (85%) of these tumors are adenocarcinomas arising from the ductal epithelium. Pancreatic cancer is rarely associated with paraneoplastic syndromes, although sensory peripheral neuropathies, cutaneous manifestations such as cicatricial and bullous pemphigoid, and Trousseau’s syndrome or migratory thrombophlebitis are known manifestations.

More commonly, pancreatic cancer will lead to hepatic panel abnormalities. Both cholestatic and hepatocellular patterns of dysfunction are seen but typically as a result of either hepatic metastasis, mechanical obstruction of a bile duct, or medication-induced injury. Hepatic panel abnormalities in the absence of these findings, a condition known as Stauffer’s syndrome, has been described as a paraneoplastic phenomenon classically in early stage renal cell carcinoma (RCC) [1-2]. Although Stauffer's syndrome has been anecdotally described with other cancers such as prostate cancer [3] and bladder cancer [4], this syndrome has never been appreciated with pancreatic cancer. Below, we describe a case of apparent Stauffer’s syndrome in a patient with pancreatic cancer.

## Case presentation

This is a 75-year-old male patient who presented with dark urine, clay-colored stool and 20 pounds weight loss. His medical history included hyperlipidemia, new onset type 2 diabetes, and past history of prostate cancer diagnosed in 2010, treated with radiation and androgen deprivation therapy. He was a professor of music and played the trumpet.

His initial physical examination, including abdominal examination was normal. The initial laboratory data showed normal white blood cells (WBC), hemoglobin (Hgb) of 10.0 g/dL and platelets of 129/cmm, with normal hepatic panel and serum chemistries. A computer tomography (CT) scan showed a mass in the head of the pancreas, which was believed to be resectable. He underwent an uncomplicated Whipple procedure with lymph node dissection yielding 23 disease-free nodes. His initial staging was T3 N0 M0, hence stage IIA disease.

He was started on adjuvant chemotherapy, consisting of gemcitabine (1000 mg/m2 D1, 8, 15 every 28 days for six months). At the time of initiation of chemotherapy, his hepatic panels were normal. Cycle one of gemcitabine was complicated by thrombocytopenia, anemia, and cellulitis requiring antibiotics. On day 1 of cycle 2, he was noticed to have hepatic panel abnormalities (Table 1). His coagulation profile—prothrombin time, partial thromboplastin time and international normalized ratio (PT, PTT, and INR)—remained within normal limits and serum albumin within the normal range. This was initially believed to be antibiotic-induced, either from linezolid and doxycycline received for treatment of cellulitis, or from pravastatin. The offending medications were discontinued and the hepatic panel normalized. However, on cycle 3 day 2, the hepatic panel became elevated again.

**Table 1:** Hepatic panel abnormalities in our patient AST: Aspartate aminotransferase (normal range: < 40 units/liter), ALT: Alanine transaminase (normal range: < 55 units/liter), ALP: Alkaline phosphatase (normal range: < 115 units/liter), Total bilirubin (normal range: 0.0-1.0 mg/dl), Direct bilirubin (normal range: 0.0-0.4 mg/dl).

Cycle (C) and Day (D)	AST (units per liter)	ALP (units per liter)	ALT (units per liter)	Total bilirubin (mg/dl)	Direct bilirubin (mg/dl)
Pre-chemo	25	85	26	0.6	
Gemcitabine C2 D1	103	134	124	0.5	
C2 D8	33	117	45	0.4	
C3 D2	419	194	301	1.4	0.8
C3 D9	48	187	143	0.5	
C3 D21	29	123	40	0.7	
5FU/LV C1 D9	180	282	467	1.3	
C1 D10	81	203	297	1.1	0.5

Workup for other liver etiologies including hepatitis and alcoholism were ruled out. In acute alcoholic hepatitis, aspartate aminotransferase (AST) is typically in the 100 to 200 IU/L range, even in severe disease, and the alanine aminotransferase (ALT) level may be normal, even in severe cases. The AST level is higher than the ALT level, and the ratio is greater than 2:1 in 70% of patients. A ratio greater than three is strongly indicative of alcoholic hepatitis. Moreover, the degrees of bilirubin level increase and prothrombin time elevation are better indicators of severity of disease. Hepatitis B serology showed only anti-HBs antibody, confirming immunization against the virus. Similarly, the patient was found to have negative anti-HCV antibody and anti-HAV IgM, ruling out both hepatitis C and A infections respectively.

Hence no etiology was recognized, and it was decided to switch from gemcitabine to 5-fluorouracil/leucovorin (5-FU/LCV) both intravenously (Days 1-5 every 28 days) for three months to rule out the possibility of gemcitabine being the culprit medication and also knowing the data from studies showing similar survival in adjuvant setting of pancreatic cancer [5-6]. On cycle 1 day 9, he presented with oral ulcers, diarrhea and recurrent elevated hepatic panel. His chemotherapy was held and both his hepatic panel and symptoms rapidly normalized. Magnetic resonance imaging (MRI) of abdomen showed no liver or biliary process. Liver biopsy at this time showed mild steatosis, acute cholangitis and pericholangitis, mild hepatocellular cholestasis, mild portal edema with moderate chronic inflammation, and sinusoidal dilatation, believed to be chemotherapy-associated steatohepatitis or an atypical Stauffer’s syndrome.

Due to kidney-based metabolism of capecitabine, he was switched from 5-FU/leucovorin to capecitabine (Xeloda) at a lower dose of 1000 mg twice a day. He continued to show periodic elevation of hepatic panel. Unfortunately, towards the end of the sixth month, he was found to have pulmonary nodules and the biopsy was consistent with metastatic pancreatic cancer. CT and positron emission tomography scan (PET) at that stage were still negative for any liver metatstasis and also there was no radiological manifestation of other diseases. He was then offered modified FOLFOX regimen consisting of oxaliplatin, 5-FU and leucovorin (m-FOLFOX). Unfortunately, before his second cycle of chemotherapy, he sustained a fall with head injury leading to brain hemorrhage and ultimately death. No biopsy was performed.

## Discussion

We believe that our case is the first patient with pancreatic cancer who manifested with Stauffer’s syndrome. Diagnostic imaging and biopsy failed to show any metastases, similar to previously described cases of Stauffer’s syndrome, which showed abnormal hepatic panel without radiographic or microscopic evidence of metastatic disease in the liver or bile ducts. The biggest supporting fact is that despite changing chemotherapeutic agents with different targets as well as different metabolic pathways (hepatic versus kidney), the hepatic panel continues to fluctuate.

Typical Stauffer’s syndromepresents elevations in the enzymes aspartate transaminase (AST) and alanine transaminase (ALT) and alkaline phosphatase (ALP) without jaundice. However, atypical presentations with a jaundice component have also been described [7-8]. The workup, which typically consists of endoscopic retrograde cholangiopancreatography (ERCP), abdominal ultrasound, and magnetic resonance cholangiopancreatography (MRCP), shows no intrahepatic or extrahepatic ductal dilation. Viral and rheumatologic studies return negative. Biopsy typically shows cholestasis, sometimes with a mild inflammatory infiltrate. Surgical removal of the otherwise local cancer via nephrectomy leads to resolution of the hepatic abnormalities [9]. In cases where there was a recurrence of cancer, this was heralded by the resurgence of the liver enzymes [2]. Table 2 summarizes the previously published cases.

**Table 2:** Previously reported cases of Stauffer's syndrome AST: Aspartate aminotransferase, ALT: Alanine transaminase, ALP: Alkaline phosphatase.

Reference	AST (U/L)	ALP (U/L)	ALT (U/L)	Total bilirubin (mg/dL)	Direct bilirubin (mg/dL)
4	1124	159	962	1.32	0.95
3	50	4679	55	18.1	9.6
8	132	193	334	2.8	2.2
	158	163	426	6.2	4.2
7	57	367	50	22.9	16.6

The pathogenesis of Stauffer’s syndromeis still incompletely elucidated. Blay, et al. found elevated interleukin-6 (IL-6) levels in patients with RCC and elevated alkaline-phosphatase, bilirubin, and gamma gluconorase transferase (GGT), suggesting an IL-6 mediated process [10]. Further investigation in this area is warranted.

Due to the incurable nature of our patient’s malignancy, it was impossible to correlate the removal of his cancer with the resolution of liver function tests (LFTs). Despite this lack of confirmation, our case illustrates the importance of keeping paraneoplastic phenomena as the differential for transaminitis. Given that there are documented cases of Stauffer’s syndrome as the presentation of RCC, this should also be included in the list of differential diagnosis of new and/or abnormal hepatic panel.

## Conclusions

In summary, Stauffer’s syndrome, a paraneoplastic elevation in hepatic panel abnormalities, is classically seen with renal cell carcinoma but can occur in other cancers including prostate cancer and now, we present the first patient with pancreatic cancer. This finding has been described as the presenting symptom of new malignancy, highlighting its place in the differential diagnosis of abnormal hepatic panel for oncologists and physicians alike.
